# Chitosan Hydrogels for Chondroitin Sulphate Controlled Release: An Analytical Characterization

**DOI:** 10.1155/2014/808703

**Published:** 2014-12-31

**Authors:** Annalisa Bianchera, Enrico Salomi, Matteo Pezzanera, Elisabeth Ruwet, Ruggero Bettini, Lisa Elviri

**Affiliations:** Department of Pharmacy, University of Parma, Parco Area delle Scienze 27/A, 43124 Parma, Italy

## Abstract

This paper provides an analytical characterization of chitosan scaffolds obtained by freeze-gelation toward the uptake and the controlled release of chondroitin sulphate (CS), as cartilage repair agent, under different pH conditions. Scanning electron microscopy (SEM), attenuated total reflectance-Fourier transform infrared spectroscopy (ATR-FTIR), and liquid chromatography-UV spectrophotometry (LC-UV) techniques were exploited to obtain qualitative and quantitative descriptions of polymer and drug behaviour in the biomaterial. As for morphology, SEM analysis allowed the evaluation of scaffold porosity in terms of pore size and distribution both at the surface (Feret diameter 58 ± 19 *μ*m) and on the cross section (Feret diameter 106 ± 51 *μ*m). LC and ATR-FTIR evidenced a pH-dependent CS loading and release behaviour, strongly highlighting the role of electrostatic forces on chitosan/chondroitin sulphate interactions.

## 1. Introduction

Despite great progresses in orthopaedics, cartilage defects still constitute a major medical issue leading to serious decrease in the quality of life and to high medical costs. This is mainly due to the fact that articular cartilage is a tissue subject to intensive wear but endowed with modest regeneration potential. Strategies for cartilage repair include the local or systemic administration of growth factors, surgery, and cell transplantation but none of them results in satisfactory cartilage healing [1, 2].

Tissue engineering is a promising field of research that relies on the interaction of three main elements, namely, a supportive material, growth factors, and cells for the replacement of damaged tissues and organs [3].

**Figure 1:**
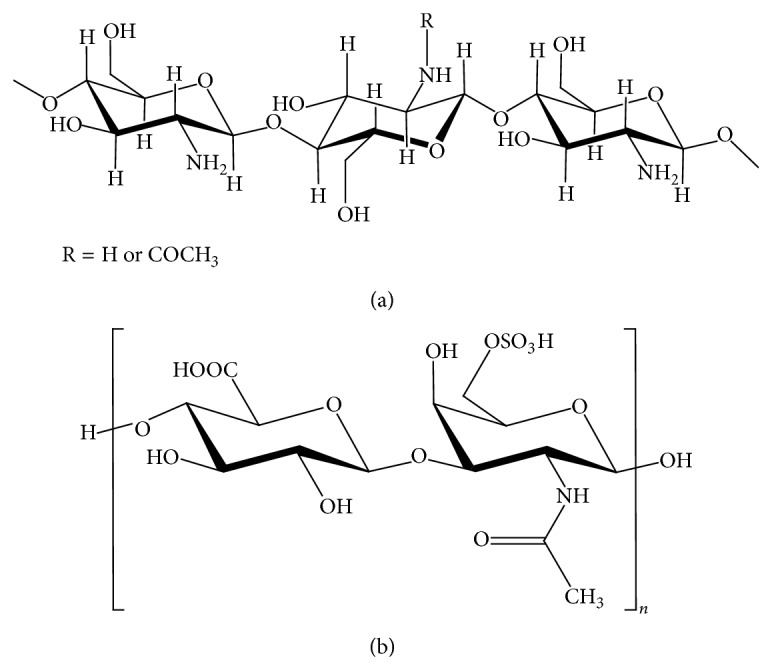
Scheme of chitosan (a) and chondroitin sulphate (b) structure.

Biomaterials suitable for tissue engineering must satisfy some requirements such as biocompatibility and biodegradation and they should act as a good substrate for cell growth. Chitosan is a polymer of natural origin deriving from the alkaline N-deacetylation of chitin (Figure 1(a)). It possesses a good combination of biocompatibility and biodegradability, it is not toxic and not expensive, and it can be moulded into any desired shape, thus making it suitable for many applications [4, 5]. Chitosan has already been used as drug carrier and is reported to participate in wound healing; moreover its structural similarity with naturally occurring glycosaminoglycans suggests chitosan as an ideal candidate for the production of scaffolds for cartilage regeneration [6, 7].

In this work, hydrogels intended for the substitution and repair of damaged cartilage were prepared by associating chitosan with chondroitin sulphate (CS). Apart from its technological features, chitosan was chosen as main component of the scaffold for both its supportive role and its intrinsic potential in helping articular cartilage repair: actually chitosan is reported to be a good support for chondrocyte cells in vitro [8, 9]. Moreover chitosan induces the expression of cartilage extracellular matrix (ECM) proteins by human chondrocytes [10] and can drive the differentiation of human and murine mesenchymal stem cells towards the chondrogenic lineage [11, 12]. Furthermore glucosamine, the basic unit of chitosan that could be released as its degradation product, can enter as a building block in the synthesis pathways of hyaluronan and peptidoglycan; this molecule and its derivatives are acknowledged to play a role in chondroprotection and to promote chondrogenic phenotype in both chondrocytes and mesenchymal stem cells [13, 14]. For these reasons glucosamine is included in the list of symptomatic slow acting drugs in osteoarthritis (SYSADOA) and structure/disease modifying antiosteoarthritis drugs (S/DMOAD) in the treatment of osteoarthritis (OA).

The application of chitosan to cartilage tissue engineering can be further improved by its association with other polymers, such as, among others, chondroitin sulphate [15]. Chondroitin sulphate (Figure 1(b)) is a dominant polysaccharide in mature cartilage in which it plays both a metabolic and a mechanical role. The presence of carboxyl and sulphate groups gives a net negative charge to this molecule in biological milieu, thus conferring to cartilage a whole fixed charge density. This charge generates a fluid influx into cartilage that guarantees its swelling and tone, in balance with the elastic restraint of collagen network. Chondrocytes can bind to chondroitin sulphate through the hyaluronan receptor CD44 and this interaction induces the mRNA expression of type 2 collagen and aggrecan [16, 17]. At clinical level, chondroitin sulphate has a chondroprotective role and is administered as a SYSADOA and S/DMOAD [18–20]. The benefits of CS for the treatment of OA are supposed to occur through three main mechanisms: (1) CS increases the synthesis of hyaluronan, glucosamine, and collagen type II by chondrocytes [21], (2) it inhibits cartilage degeneration [22] by ECM degrading enzymes [23], and (3) it has an anti-inflammatory effect by suppressing inflammatory mediators [24]. These protective effects on chondrocytes are further potentiated by the association of CS to glucosamine [25].

As reported by Sechriest et al. [26], chitosan films covered by a layer of CS promoted the adhesion and growth of chondrocytes which maintained their morphological and functional features in vitro. For these reasons the association of chitosan and chondroitin sulphate is supposed to offer a good support for adhesion and growth of cells, desirably driving them towards the generation of a healthy hyaline cartilage. Furthermore, chondroitin sulphate locally released in the site of cartilage damage could exert a significant anti-inflammatory, anticatabolic, and antiangiogenic effect [27, 28], contributing to the restraint of the inflammatory state.

From a chemical point of view, chitosan and chondroitin sulphate possess opposite charges that almost completely hamper the preparation of blend solutions. For this reason, the techniques applied so far in order to get their association involve the chemical modification of chitosan to improve its solubility characteristics [29], ionic or chemical cross-linking [30–33], the formation of polyelectrolyte complexes followed by lyophilisation or air drying [34–39], or a combination of those methods [40–42]. So far, the preparation of blend solutions of both chitosan and chondroitin sulphate was reported only by Yao et al. [43], with a relatively low concentration of chitosan and a very high chitosan to chondroitin sulphate final weight ratio (55 : 1); the solid state characteristics of the membranes were analysed but nothing was reported about their behaviour in terms of chondroitin sulphate release kinetic. A detailed characterization of chondroitin sulphate release from chitosan membranes was reported by Piai et al. [44], taking into consideration different pH conditions.

The development of innovative biomaterials for clinical uses presents transdisciplinary aspects including analytical studies. In these aspects, the use of different analytical techniques is a demand for the understanding of the properties of the biomaterial. Here we report the analytical characterization of chitosan scaffolds prepared for the targeted and controlled delivery of chondroitin sulphate.

Since surface properties affect the success or failure of the scaffold device, scanning electron microscope (SEM) technique was used for surface and cross section subsequent characterization. Attenuated total reflectance-Fourier transform infrared (ATR-FTIR) and liquid chromatography-UV spectrophotometry (LC-UV) were complementarily used for scaffold characterization and CS loading and release evaluation. In reference to the analytical results, the nature of the interaction between the two molecules and the behaviour of scaffolds at different pH conditions were discussed for both CS uptake and CS release.

## 2. Experimental

### 2.1. Reagents

Chitosan fine powder (deacetylation degree = min 90%), chondroitin sulphate from shark cartilage, and KOH were purchased from A.C.E.F. (Piacenza, Italy); raffinose pentahydrate was from Sigma-Aldrich. All other reagents of analytical grade were obtained from Sigma Chemical Co. (St. Louis, MO, USA).

### 2.2. Chitosan Purification

Chitosan was purified by alkaline precipitation. Briefly, a 2% w/v chitosan solution was prepared in 1% w/v acetic acid aqueous solution and stirred to complete dissolution. A 3% w/v KOH aqueous solution was prepared in order to have half volume of the chitosan solution and added drop by drop at a rate of 60 drops/min to induce chitosan precipitation. The obtained dispersion was filtered through filter paper on a Buckner funnel and then rinsed three times with 96% ethanol. The resulting slurry was then transferred to an oven set at 40°C and dried. The resulting yellowish powder was grinded and sieved to 600 mm sized particles.

### 2.3. Scaffold Preparation

Scaffolds were prepared according to the method reported by Lippiello [25]: a 4.5% chitosan solution was prepared by dissolving purified chitosan in a 1% acetic acid aqueous solution and then raffinose pentahydrate was added at a final concentration of 290 mM as viscosity modifying agent. After complete dissolution, the solution was cast into 10 mm diameter rubber rings and frozen at −60°C overnight. Frozen scaffolds were then transferred in a cold gelation solution made of four parts of a KOH 5% aqueous solution and six parts of 96% ethanol and left to gel at −20°C for 24 hours. Scaffolds were then rinsed in double distilled water and kept in water until chondroitin sulphate loading.

### 2.4. SEM Analysis

Freshly prepared scaffolds were dehydrated in a graded series of ethanol solutions (from 70 to 99.8% v/v) and then air-dried. Images were taken with a scanning electron microscope (Sigma HD, Carl Zeiss, Jena, Germany) and analysed by ImageJ64 software (NIH, USA) for pore size determination (Feret diameter), distribution, and pore interconnectivity. The average pore dimension was calculated on a 500 *μ*m^2^ surface area and expressed as median diameter, D50, value.

### 2.5. ATR-FTIR Characterization

In order to identify the kind of interactions occurring among chitosan and chondroitin sulphate, ATR-FTIR experiments were carried out by mixing chitosan or dried scaffolds with chondroitin sulphate at two different ratios (chitosan : CS, 5 : 2 and 1 : 1 w/w). Spectra were collected with a Thermo Nicolet 5700 spectrometer equipped with a Thermo Smart Orbit ATR diamond accessory. The scanning wavenumber range was 400–4000 cm^−1^ with a resolving power of 2 cm^−1^.

### 2.6. Chondroitin Sulphate Loading and Release

Loading of chondroitin sulphate was performed at 25°C by immersing chitosan scaffolds into a 1 mg mL^−1^ chondroitin sulphate solution in 10 mM phosphate buffer adjusted at pH 4.5, 6, or 8 with NaOH or HCl solution. The amount of loaded CS was calculated by regularly sampling the solution (sampling volume: 0.5 mL) over eight days and measuring the amount of chondroitin sulphate left in solution by LC-UV. Aliquots of sampled solution were replaced with purified water after each collection. Loading time was one week.

As for release, a Franz-type diffusion cell with a porous (0.45 *μ*m) regenerated cellulose membrane as solid barrier between the donor and receptor compartment was used: as both donor and receiving solution a 10 mM phosphate buffer at pH 7.4 and 37°C was used. Release experiments were performed up to 4 days.

The stability of chitosan scaffolds and chondroitin sulphate was checked by LC-UV in each working condition for the duration of the whole experiment.

### 2.7. Chondroitin Sulphate Quantitation by LC-UV

A LC-UV method was developed by using an HP 1200 liquid chromatograph (Agilent Technologies, Santa Clara, CA, USA) equipped with and autosampler and an UV detection system. The mobile phase was MilliQ water delivered at a flow of 0.45 mL min^−1^ onto a C18 (20 × 2.1 mm, 5 *μ*m) cartridge (Phenomenex, Torrance, CA, USA); 50 *μ*L of each sample was injected five times. The amount of chondroitin sulphate loaded and released by the scaffolds was quantified by monitoring the signal of UV absorbance at a wavelength of 210 nm. The method was validated following ICH guidelines, for the quantification of chondroitin sulphate as well as for the evaluation of stability of CS solutions. In particular, detection limits (LODs), quantitation limits (LOQs), linearity, precision, and selectivity were calculated as follows: LOD = 3.3*σ*/*b* and LOQ = 10*σ*/*b*, where *σ* is the standard deviation of five blank (aqueous solution at different pH) measurements and *b* is the slope of a calibration curve. Linearity was evaluated over two orders of magnitude in the 0.01–1 mg mL^−1^ concentration range, by analysing three replicated injections at five levels. Precision was evaluated in terms of repeatability on five replicated injections at three concentration levels and interday precision on five replicated injections at three concentration levels on three different days.

## 3. Results and Discussion

### 3.1. Scaffold Preparation and Characterization

Chitosan scaffolds were prepared as described in Experimental section and characterized by SEM and ATR-FTIR analysis.

**Figure 2:**
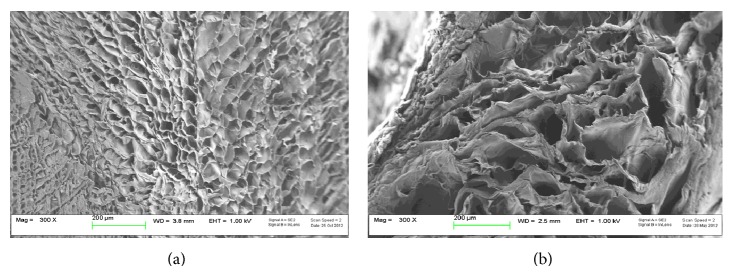
Morphological characterization of chitosan scaffold. SEM images of chitosan scaffold (a) surface and (b) internal cross section.

As for morphology, SEM images of the chitosan scaffold exhibited optimal surface homogeneity in terms of pore size and distribution (Feret diameter 58 ± 19 *μ*m) (Figure 2(a)). The cross-sectional micrographs revealed a regular interconnected and layered pore structure (Feret diameter 106 ± 51 *μ*m) in the interior region (Figure 2(b)).

**Figure 3:**
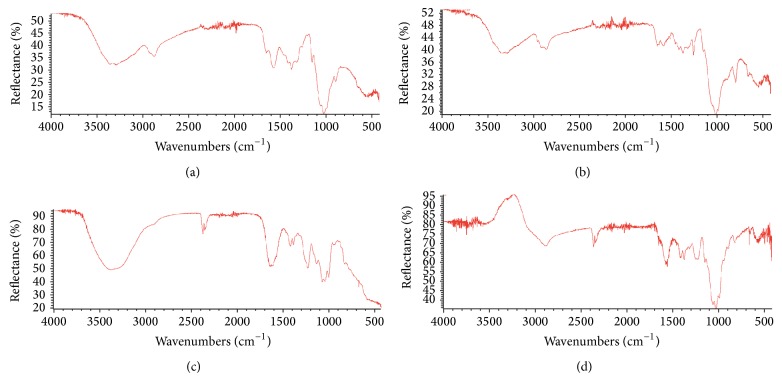
ATR-FTIR spectra of (a) chitosan, (b) chitosan scaffold, (c) chondroitin sulphate, and (d) chitosan scaffold loaded with CS.

Initially, ATR-FTIR analysis was performed on the chitosan powder used for scaffold preparation. Characteristic bands of chitosan were evident in the spectrum at 3400 cm^−1^ (–OH group), 1650 cm^−1^ (–C=O stretching), 1595 cm^−1^ (N–H bending vibration), and 1380 cm^−1^ (–C–O stretching of primary alcoholic group), respectively. The O=C–NH band was slightly visible at 3300 cm^−1^ (Figure 3(a)).

Significant changes in the spectrum of chitosan scaffold were observed with respect to that of chitosan powder. In particular, bands at 3400 cm^−1^ (–OH group), 1655 cm^−1^ (N–H bending vibration), and 1380 cm^−1^ (–C–O stretching of primary alcoholic group) decreased or disappeared suggesting a significant role of these functional groups in establishing the tridimensional and supramolecular structure of the scaffold. Significant changes were observed also in the region between 800 and 1200 cm^−1^. In detail, strong bands appeared at 1260 cm^−1^, 1095 cm^−1^, and 1020 cm^−1^ (Figure 3(b)).

Chondroitin sulphate was analysed as raw powder as well and the spectrum showed the characteristic bands at 3280 cm^−1^ (–OH group), 3100 cm^−1^ (–N–H stretching), 1660 cm^−1^ (–C=O stretching), 1560 cm^−1^ (N–H bending vibration), and 1240 cm^−1^ (R–OSO_2_–O^−^) (Figure 3(c)).

The mixture of chitosan and chondroitin sulphate afforded a decrease of the R–OSO_2_–O band of CS at 1240 cm^−1^ and the disappearance of the 1595 cm^−1^ (N–H bending vibration) and the 1320 cm^−1^ bands of chitosan (Figure 3(d)). These data suggest that these functional groups are strongly involved in an interaction between the two macromolecules.

### 3.2. Validation of the LC-UV Method

For quantitation of chondroitin sulphate during uptake and release experiments, as well as stability test, a fast and reliable LC-UV spectrophotometric method was developed and validated. A rigorous validation procedure following ICH guidelines [26] was followed. In particular, the method was validated in terms of detection limits, quantitation limits, linearity, precision, and selectivity. In the validation of a method developed for the study of loading and release experiments it is important to define the lowest concentration of analyte that can be detected and quantified with known precision. Good LOD and LOQ values were obtained, 3.9 and 12 *μ*g/mL, respectively. Linearity was explored starting from the LOQ value over two orders of magnitude [*Y* = 15025(±9)*X*; *r* ^2^ = 0.999] and excellent determination coefficient was obtained. Method precision was calculated in terms of intraday repeatability and intermediate precision. As shown in Table 1, RSD% values lower than 3% were indicative of excellent repeatability on three concentration levels. Results from the homogeneity test performed on the experimental data acquired over three days evidenced that method precision is constant (Table 1). As for selectivity, since no interfering signals were detected by analysing blank samples, excellent selectivity was found under the operative conditions used.

### 3.3. Chitosan Scaffold Loading and Release Behaviour

Loading and release experiments were usually carried out over a week; therefore, establishing stability of chondroitin sulphate and chitosan scaffold under operative conditions was a mandatory prerequisite. For this reason the stability of both chondroitin sulphate and chitosan scaffold was evaluated in aqueous solution at different pH by LC-UV analysis. Data were collected on seven days performing one sampling and five replicated injections per day. An analysis of variance was firstly carried out before comparing means by a *t*-test. Results for CS exhibited a nonsignificant difference (*P* > 0.05) among group variances but a significant difference among mean values with a reduction of about 10% nominal concentration over the time explored for all the pH values tested.

**Figure 4:**
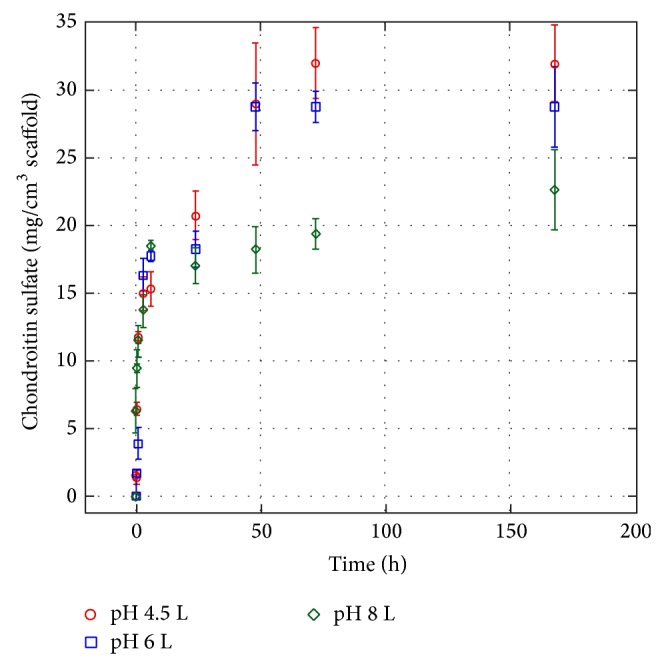
Cumulative chondroitin sulphate loading on the chitosan scaffold exposed at different solution pH (10 mM buffer concentration).

In a further step, we investigated the capability of chitosan scaffolds to load CS from aqueous solutions at three different pH values (i.e., pH 4.5, 6, and 8). Very interestingly, chitosan scaffolds prepared in this work were able to load large amounts of CS (up to approximately 30 mg cm^−3^). The loading profiles reported in Figure 4 show statistically significant effects of pH on CS loading (*P* < 0.05) into chitosan scaffolds. In particular, the amount of CS loaded increased as the pH decreased. It is well known that since chitosan carries ionisable groups, its properties depend, besides its degree of deacetylation and molecular weight, on pH and ionic strength of solution. The p*K*_a_ value of glucosamine units ranges between 6.3 and 7. During scaffold preparation, pH varies from acidic (1% acetic acid) to basic (5% KOH) values, with a consequent increase and decrease of chitosan charge state, respectively. Chitosan presents a relatively good conformational flexibility, only limited by the bulky sugar moiety, allowing a spatial arrangement as a function of electrostatic repulsion forces. During scaffold assembly, the pH of solution, below 5, should allow the exposure of protonated amine groups by electrostatic repulsion: this conformation is supposed to be retained during gelation at basic pH because the presence in the solution of raffinose at high concentration determines a consistent increase in viscosity that results in a reduced molecular mobility of chitosan chains.

This hypothesis is supported by data collected at pH 4.5 and 6 which indicate an increase of CS-chitosan affinity at lower pH, suggesting that strong electrostatic interactions can occur between the highly positively charged NH_3_^+^ groups on chitosan and the negatively charged SO_3_^−^/COO^−^ groups on CS.

These hypotheses are supported even by the partition coefficient values observed for the scaffolds loaded with CS under the different operative conditions. The partition coefficient at equilibrium as a function of pH was calculated according to the following formula:

(1) $$\begin{array}{c} k=\frac{{\left[\text{CS}\right]}_{\text{scaff}}}{{\left[\text{CS}\right]}_{\text{sol}}}, \end{array}$$

where [CS]_scaff_ is the concentration of CS loaded into scaffold at equilibrium and [CS]_sol_ is the concentration of CS left in solution at equilibrium. Analogous results were obtained at pH 4.5 and 6.0 (*k* = 41), whereas at pH 8.0 a significant reduction was observed (*k* = 26).

**Figure 5:**
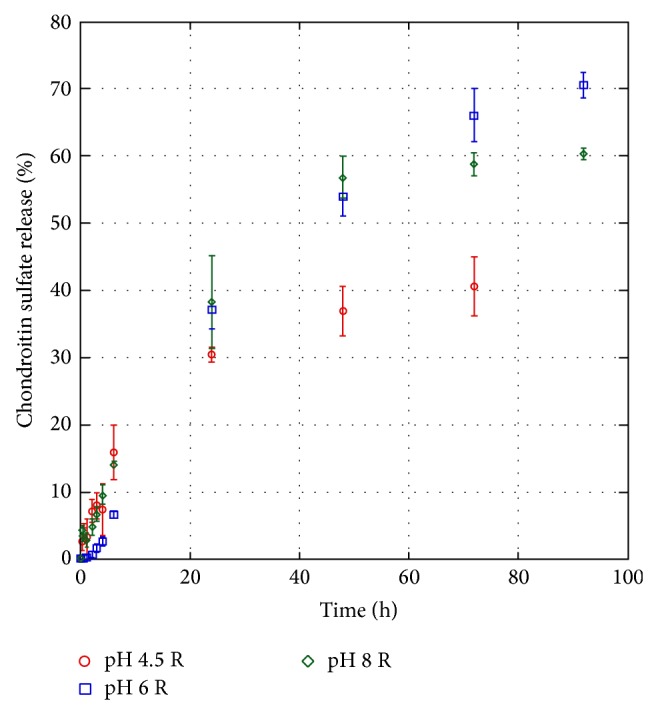
Condroitin sulphate percent release from condroitin sulphate-chitosan scallold as a function of time in phoshate buffer solution (50 mM, pH 7.4).

Finally, the release of chitosan from loaded scaffolds was investigated using as a dissolution medium a phosphate buffer, 50 mM, pH 7.4 (Figure 5). A very similar release profile was observed for scaffolds loaded at pH 6 and 8, respectively. Up to approximately 70% of the loaded CS was released in 92 hours. Release data obtained from the chitosan scaffold loaded at pH 4.5 indicated a higher retention of CS (40% release in 72 hours). Since all release experiments were carried out in the same medium (phosphate buffer, 50 mM, pH 7.4), the differences in release profile could still reflect a different partitioning and distribution of CS in chitosan scaffolds during the loading phase. In fact, at pH 4.5 chitosan/CS interactions could be favoured by the synergistic effect of intermolecular attractive forces, due to opposite charged groups of chitosan and CS, and of intramolecular repulsive forces due to positively charged amine groups in chitosan that could offer an easier accommodation to CS molecules inside the polymeric network of the scaffold. This effect is less pronounced at pH 6 and 8 due to the change in ionization state. When scaffold is exposed to pH 7.4, residual charges are further neutralized determining a tightening of chitosan network and a consequent entrapment of CS molecules within it.

## 4. Conclusions

In this work the application of appropriate analytical techniques allowed a deep characterization of the properties of chitosan-based scaffolds. Such scaffolds were able to load useful amounts of chondroitin sulphate, up to 30 mg cm^−3^. A pH-dependent loading behaviour was observed, strongly evidencing the role of electrostatic forces on chitosan/chondroitin sulphate interactions.

As for release, it was interesting to evidence the capability of these scaffolds to perform a controlled release of CS. Release experiments performed at pH 7.4 (above p*K*_a_ of glycosaminoglycan structure) resulted in a common initial burst release, independent of the pH of loading, probably due to the presence of CS on scaffold surface readily available for the contact with solvent, followed by a release behaviour that varied as a function of loading conditions. In particular, for scaffolds loaded at pH 4.5 the amount of CS released is significantly lower than the amount released from scaffolds loaded at pH 6 and 8. Loading pH conditions were supposed to be able to change scaffold pore size playing an important role in CS diffusion in cooperation with ionic interactions for the uptake in the whole scaffold region.

## Figures and Tables

**Table 1:** Repeatability and intermediate precision of the LC-UV method.

	Level I (0.01 mg/mL)	Level II (0.3 mg/mL)	Level III (1 mg/mL)
	Mean value ± SD (RSD %)		
Repeatability	222.35 ± 7.15 (3.22)	4122.11 ± 28.91 (0.70)	12530.9 ± 33.06 (0.26)
Intermediate precision	248 ± 22 (8.83) (*P* = 0.27)^a^	4974 ± 55 (1.12) (*P* = 0.99)^a^	15208 ± 153 (1.01) (*P* = 0.09)^a^

^a^Homogeneity of variance test: confidence level, 95%.
